# Kinesio taping for ankle sprain in youth athlete: A protocol for systematic review and meta-analysis

**DOI:** 10.1097/MD.0000000000031222

**Published:** 2022-10-21

**Authors:** Nan Yang, Shan Chen, Kui Cui, Li Li

**Affiliations:** a Shanghai University of Sport, Shanghai, China; b Physical Education Teaching and Research Office, High School Affiliated to Fudan University, Shanghai, China; c Physical Education Teaching and Research Office, Yangpu District Education Institution of Shanghai, Shanghai, China.

**Keywords:** ankle sprain, Kinesio taping, meta-analysis, systematic review, youth athlete

## Abstract

Ankle sprain is considered a major problem that may hinder youth athletes’ athletic development because it will lead to ongoing dysfunction, reoccurrence of ankle sprain, chronic ankle instability, and posttraumatic osteoarthritis. Kinesio taping (KT) is a therapeutic taping technique that has been widely used in the treatment of various ankle issues including sprained ankles and in the prevention of ankle sprains. It can not only provide the injured ankle with support during the rehabilitation phase, but also enhance the ankle stability during activity. However, the available evidence regarding its effectiveness in the treatment and prevention of ankle sprain is inconsistent. Therefore, a systematic review will help clinicians and coaches better understand the application of KT in clinical and training practices. This study is to systematically review the literature on the use of KT to improve outcomes including ankle function, proprioception, and pain and to evaluate the effectiveness of KT in the treatment and prevention of ankle sprain injuries.

**Methods:**

A comprehensive electronic search of the literature will be undertaken in the following databases: PubMed, CINAHL, SPORTDiscus, Cochrane library, Web of Science and Scopus from 1979 to August 2022. The Physiotherapy Evidence Database scale will be used to assess the methodological quality of all included studies and RevMan 5.3 (Copenhagen, The Nordic Cochrane Centre) for the data analysis.

**Results:**

This study will provide a standardized evaluation and comparison for effects of KT on the treatment and prevention of ankle sprains in youth athletes.

**Conclusion:**

This review will provide the evidence of the effectiveness of KT used in the treatment and prevention of ankle sprain in youth athletes. This review will also provide directions and recommendations for future research and clinical practices targeting treatment and prevention of ankle sprains in youth athletes.

## 1. Introduction

The ankle is the most frequently injured body part in sports.^[1]^ Up to 80% of ankle injuries are ankle sprains.^[1,2]^ Evidence indicates that ankle sprains may lead to consequences including ongoing ankle dysfunction, recurrence of ankle sprain, chronic ankle instability, and posttraumatic osteoarthritis.^[3–6]^ Valderrabano et al^[7]^ reported that 23% of the high school and Division I athletes were identified as having chronic ankle instability. Ankle sprain and its following consequences may affect youth athletes’ active participation in training and competition, and eventually hinder their athletic progression and development, especially for those at a crucial transition into higher-level physical demands. Therefore, various ways of treating and preventing ankle sprain injuries, including taping or bracing,^[8,9]^ strength training,^[9]^ and proprioceptive training,^[9]^ have been tried to help improve comfort and speed recovery in clinical trials and training practices.

Kinesio taping (KT) is a therapeutic taping technique developed by Dr Kenzo Kase in 1970s. Compared with traditional taping and bracing, KT is considered better tolerated and cost effective for the patients because it is elastic, fast drying and can be left on skin for up to 5 days.^[10]^ Clinically, it has been used as an alternative to the traditional taping in the treatment of a variety of ankle issues including sprained ankles, inflamed tendons in the ankle, stretched ligaments, or general ankle weakness. It is proposed by the manufacturer that KT can be used in ankle injuries rehabilitation and ankle sprain management by providing support to injured muscles and joints, reducing pain, altering muscle function, improving circulation, enhancing proprioception, and repositioning subluxed joints.^[11]^

However, conflicting evidence regarding the effectiveness of KT in the treatment and prevention of ankle sprains can be found in extant literature. For example, Nunes et al^[12]^ reported that the application of KT was ineffective in decreasing acute swelling after an ankle sprain while Aguilar-Ferrándiz et al^[13]^ found a reduction in swelling in the Kinesio Taping group. Lee and Lee^[14]^ treated young women with inversion and eversion sprains using kinesio tape. They identified reduced ankle instability and pain and improved functional dynamic balance after 4-week ankle taping and thus concluded that repeated ankle taping may be an effective treatment intervention for ankle inversion and eversion sprain.^[14,15]^ Nunes et al^[12]^ indicated that discrepancy in results between studies may result from the varied patient conditions considered and different taping techniques and taping time employed.

As for the effect of KT on prevention of ankle sprains, a review by Wang et al^[16]^ indicated that Kinesio taping is superior to other taping methods (athletic taping) in ankle functional performance improvement and recommended that KT be used to maintain foot and ankle mobility during rehabilitation and prevent prolonged disability and subsequent overuse injuries. However, Briem et al^[17]^ found in their study that KT had no effect on muscle activation of the fibularis longus and concluded that KT may not prevent ankle sprains by enhancing dynamic muscle support of the ankle.

As the available evidence of the effectiveness of KT is inconsistent, a systematic review is necessary to collate and compare the outcome measures following KT among youth athletes to make sure whether KT is an effective way for youth athletes to treat and prevent ankle sprain injuries. Therefore, this meta-analysis aims to systematically review randomized controlled trials that examined the effect of KT used for treatment and/or prevention of ankle sprain injuries in youth athletes and evaluate the effectiveness of KT by comparing outcome measures including pain, proprioception and ankle functional performance.

## 2. Methods

### 2.1. Study registration

This systematic review has registered on the PROSPERO system with the register number CRD42022354855. This protocol is developed and reported based on the Preferred Reporting Items for Systematic Reviews and Meta-Analysis statement.^[18]^

### 2.2. Inclusion and exclusion criteria

#### 2.2.1. Inclusion criteria.

##### 2.2.1.1. Types of studies.

Randomized controlled trials will be included in this review.

##### 2.2.1.2. Types of participants.

Studies focusing on youth athletes from 7 to 18 years old inclusive involved in organized/formal sports training and competitions will be included. Youth athletes with and without ankle sprain injuries will be of interest because the purpose of this study is to consider the use of KT in both treatment and prevention of ankle sprain. No restrictions will be applied on gender, race, or region.

##### 2.2.1.3. Types of interventions.

The intervention is defined as Kinesio taping. The control group is defined as participants receiving sham taping (e.g., Kinesio taping applied without tension), other taping methods (e.g., athletic taping) or no taping.

##### 2.2.1.4. Types of outcome measures.

The primary outcomes include pain, proprioception and ankle function (e.g., flexibility, balance, joint mobility, joint stability, muscle strength, etc.).

#### 2.2.2. Exclusion criteria.

Studies will be excluded if: population of focus was not youth athletes aged from 7 to 18; studies did not include a comparison group; studies were not written in English; or studies only have abstracts available.

### 2.3. Search methods

#### 2.3.1. Electronic search strategy.

A comprehensive electronic search of the literature will be undertaken in the following databases: PubMed, CINAHL, SPORTDiscus, Cochrane library, Web of Science and Scopus from 1979 when Kinesio Tape was invented by Dr Kenzo Kase to July 2022. Database limitations applied at the search phase were English language, peer-reviewed articles and full-text available. The keywords used in the literature search include “kinesio tape*” or “kinesio taping*” and “ankle injury” or “ankle sprain*” and “function” or “strength” or “pain” and “youth athlete*” or “school sport.” A detailed example of the PubMed search strategy is shown in Table 1.

**Table 1:** PubMed search strategy.

No.	Search term
#1	((((ankle) and (injury) OR (sprain)) OR (lateral ligament injury))OR(chronical instability))
#2	(taping) OR (kinesio taping) OR (kinesio tape)
#3	(function) OR (functional performance) OR(mobility) OR (flexibility) OR (stability) OR (balance) OR (strength)
#4	(proprioception) OR (position sense)) OR (movement detection) OR (movement discrimination)
#5	(pain) OR (swelling) OR (inflammation)
#6	((youth) OR ((child) OR ((school) and (athlete)))OR(player)))
#7	#1 AND #2 AND#3AND#4 AND #5) AND #6

#### 2.3.2. Searching other resources.

Reference lists of included studies and published reviews will be manually screened for additional potentially relevant articles.

### 2.4. Data collection and analysis

#### 2.4.1. Selection of studies.

**Figure 1.**
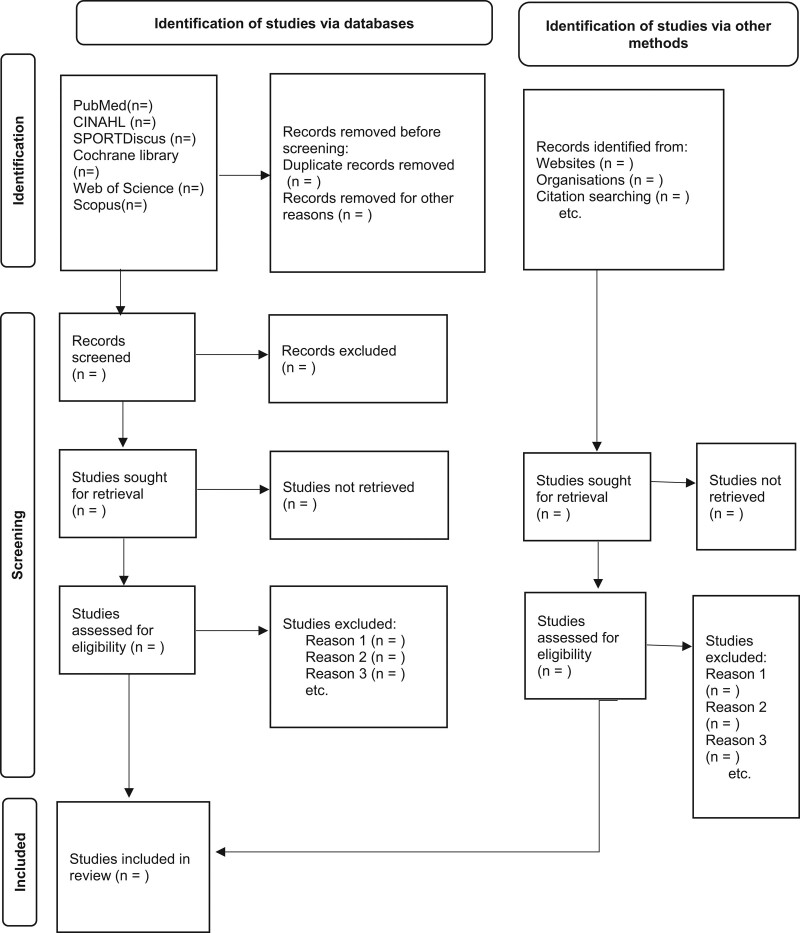
The PRISMA flow diagram of study selection process. PRISMA = preferred reporting items for systematic review and meta-analysis.

Two authors will work independently to screen the literature according to the inclusion and exclusion criteria. Then they will cross check the eligibility status of identified studies by screening titles, abstracts, and finally the full paper. In case of any disagreement, consensus will be reached through group discussions or by including a third author as a referee. The whole process of study selection is summarized in the Preferred Reporting Items for Systematic Reviews and Meta-Analysis flow diagram (adapted from Page, McKenzie, Bossuyt, Boutron, Hoffmann, Mulrow^[19]^). Screening operation is rendered in Figure 1.

#### 2.4.2. Data extraction and management.

Two authors will use a predesigned form to extract information from each eligible study. The following data will be extracted: the first author’s name; year of publication; participant demographics (age, weight, height and sex); patient conditions (acute ankle sprain, chronic ankle instability, etc.); sample size; intervention details (taping duration and taping methods); and main outcomes (ankle functional performance, pain, or proprioception). Two authors will collate the extracted data for accuracy and consistency. Disagreement will be resolved through group discussion or by including a third author as a referee.

#### 2.4.3. Risk of bias assessment.

The methodological quality of all included studies will be rated according to the guidelines of the Physiotherapy Evidence Database scale.^[20]^ Two authors will independently conduct the quality assessment process. Initial disagreements will be resolved by discussion between the two authors or by including a third author as a referee. All included studies will be classified according to the Physiotherapy Evidence Database score with the recommended reference indicator of the methodological quality (9–10 = excellent; 6–8 = good; 4–5 = fair; <4 = poor).

#### 2.4.4. Measures of intervention effect.

For all the included studies, the percentage change in the main outcomes from baseline will be calculated and compared. RevMan 5.3 software will be used to calculate effect size. For the dichotomous data, a risk ratio with 95% confidence intervals will be used to present the intervention effect, and for continuous data, mean difference or standardized mean difference with 95% confidence intervals will be used.

#### 2.4.5. Missing data management.

If data in some studies are incomplete, we will try to contact the first or corresponding author by email. If data cannot be obtained, we will analyze the available data.

#### 2.4.6. Assessment of heterogeneity.

The Higgins *I*^2^ test will be used to detect the heterogeneity of results across studies. The heterogeneity will be rated as follows: may not be important, 0% to 40%; moderate heterogeneity, 30% to 60%; substantial heterogeneity, 50% to 90%; and considerable heterogeneity, 75% to 100%.^[21]^ If *I*^2^ value <50%, a fixed effect model will be used. If *I*^2^ value >50% and *P* < .5, a random effect model and subgroup analyses will be applied.

#### 2.4.7. Subgroup analysis.

If heterogeneity over the substantial level is identified in the included studies, a subgroup analysis will be performed based on taping time (e.g. long term/short term), gender and age.

#### 2.4.8. Sensitivity analysis.

If the included studies are sufficient, sensitivity analysis will be performed to assess the robustness of studies according to methodological quality, sample size, and missing data.

### 2.5. Confidence in cumulative evidence

The evidence strength will be assessed using the Grading of Recommendations, Assessment, Development and Evaluation (GRADE). The results will be defined as the following 4 levels: high, moderate, low, and very low.

### 2.6. Ethics and dissemination

The ethical approval is not required as the data in this study will be retrieved from published research. The results will be disseminated by publishing the research in a peer-reviewed journal.

## 3. Discussion

Research has shown that 30% of all sports injuries are related to ankle complex joint, and ankle sprain ranks the most frequent in ankle injuries.^[1,22,23]^ Engebretsen et al^[24]^ point out that people who participate in team sports, contact sports, indoor sports and jumping sports are at the greatest risk of injury. And a review by Doherty et al^[25]^ reveals that females, children and adolescents report a higher incidence of ankle sprains compared with males and adults. Therefore, as youth athletes may sustain greater risk for ankle sprain, treating and preventing ankle sprain injuries are of great importance to their athletic development.

As a therapeutic taping technique, Kinesio taping has widely used in treatment and prevention of ankle sprains by giving support to injured muscles and joints and facilitating rehabilitation of sprained or strained ankles. However, the effect of KT for treatment and prevention ankle sprain are still uncertain, so this systematic review was to investigate whether there are sufficient data to support the use of KT in youth athlete with or without ankle sprains. The findings of this meta-analysis will provide meaningful implications for clinical and training practices.

## Author contributions

**Conceptualization:** Nan Yang, Shan Chen.

**Data curation:** Nan Yang, Shan Chen, Kui Cui, Li Li.

**Formal analysis:** Nan Yang, Shan Chen, Kui Cui, Li Li.

**Funding acquisition:** Nan Yang.

**Methodology:** Nan Yang, Shan Chen, Li Li.

**Software:** Nan Yang, Shan Chen, Kui Cui.

**Supervision:** Nan Yang.

**Writing – original draft:** Nan Yang.

**Writing – review & editing:** Shan Chen, Nan Yang.
